# Public Data Archiving in Ecology and Evolution: How Well Are We Doing?

**DOI:** 10.1371/journal.pbio.1002295

**Published:** 2015-11-10

**Authors:** Dominique G. Roche, Loeske E. B. Kruuk, Robert Lanfear, Sandra A. Binning

**Affiliations:** 1 Division of Evolution, Ecology and Genetics, Research School of Biology, The Australian National University, Canberra, Australian Capital Territory, Australia; 2 Éco-Éthologie, Institut de Biologie, Université de Neuchâtel, Neuchâtel, Switzerland; 3 Institute of Evolutionary Biology, School of Biological Sciences, University of Edinburgh, Edinburgh, United Kingdom; 4 Department of Biological Sciences, Macquarie University, Sydney, Australia

## Abstract

Policies that mandate public data archiving (PDA) successfully increase accessibility to data underlying scientific publications. However, is the data quality sufficient to allow reuse and reanalysis? We surveyed 100 datasets associated with nonmolecular studies in journals that commonly publish ecological and evolutionary research and have a strong PDA policy. Out of these datasets, 56% were incomplete, and 64% were archived in a way that partially or entirely prevented reuse. We suggest that cultural shifts facilitating clearer benefits to authors are necessary to achieve high-quality PDA and highlight key guidelines to help authors increase their data’s reuse potential and compliance with journal data policies.

## Abstract 2

Careful scrutiny of one hundred studies in journals that mandate data archival reveals that over half do not comply with their journal’s data policy.

Mandated public data archiving (PDA) is becoming the norm for leading journals in many fields, including ecology and evolution. Funding agencies, researchers, and publishers increasingly recognize that research articles are not the only product of scientific investigation, and greater value is being placed on the underlying data. PDA has numerous benefits for the scientific and wider community (sensu [1,2–5]), namely by allowing research results to be reproduced and data to be reused [6–8], which maintains both scientific rigor and public confidence in science [5,9,10]. Similarly, sharing data accelerates scientific discoveries and saves taxpayers’ money by avoiding unnecessary duplication of data collection [3,7,11–13].

Despite the obvious benefits of PDA for science, many researchers remain reluctant to share their data publicly [1,3,12,14–19]. This reluctance probably stems from concerns about competition for publications based on shared data, the time necessary to prepare files for archiving, a lack of recognition for PDA, and concerns about data misinterpretation [1,19, 20]. As such, perceived costs to individual researchers or research projects might offset potential group benefits for the scientific community [1,21]. To increase archiving rates, many journals have therefore resorted to strong policies including mandatory PDA. These policies work. For example, a recent review of studies in population genetics showed that implementing a PDA policy requiring a data availability statement in the published manuscript increases PDA nearly 1,000-fold [22], and an evaluation of phylogenetic studies found that data are more likely to be deposited in online archives if the journal has a strong PDA policy [23].

Making data publicly available is, however, only one requirement of PDA policies, the core aim of which is to allow reproduction of the results in the paper [24–26]. Despite growing evidence that PDA policies ensure that something is archived, assessments of the reproducibility of scientific results are rare and, to date, restricted to genetic data. Amongst these, one recent survey of 18 microarray studies found that only two were fully reproducible using the archived data [27]. Another study of 19 papers in population genetics found that 30% of analyses could not be reproduced from the archived data and that 35% of datasets were incorrectly or insufficiently described [9]. These findings are notable given that PDA is arguably most widely accepted in areas of biology that produce genetic data [12,28]. There are many factors that can hinder reproducibility, including failure to adequately describe methods [29] or failure to archive the computer code used to clean or analyse the data [30,31]. Here, we focus on the completeness and reusability of the archived datasets themselves.

How well do (nonmolecular) experimental and observational studies in ecology and evolution (E&E) fare in comparison to molecular studies? The question is of particular interest given that (1) mandatory PDA is much more recent in these fields [12,32], (2) many E&E journals currently lacking a PDA policy are likely to implement one in the near future (e.g., [33]), and (3) some of the concerns about PDA, in particular data misinterpretation, are perceived to be particularly widespread in E&E [1,19].

To answer this question, we examined data from 100 nonmolecular evolutionary and/or ecological publications that were archived in the popular data repository Dryad (http://datadryad.org/) between 2012 and 2013, from seven leading journals that regularly publish E&E research (Table 1). These journals all have strong data archiving policies: either by implementing their own policy (i.e., close to mandatory [22,34]) or by adopting the Joint Data Archiving Policy (JDAP), which requires that “data supporting the results in the paper be archived in an appropriate public archive” [35,36]. We evaluated the quality of archived data on two counts (Fig 1, S1 Text). First, are all the data supporting a study’s findings publicly available (“completeness”), thereby complying with the journals’ archiving policies? Second, although JDAP does not explicitly require that data be archived in a way that facilitates reuse, how readily can the archived data be accessed and reused by third parties (“reusability”)? We assigned each study separate completeness and reusability scores between 1 (low) and 5 (high) (see Table 2 and S1 Text for the scoring system and S2 Text for an assessment of score agreement across different raters, which was high for both scores).

**Fig 1:**
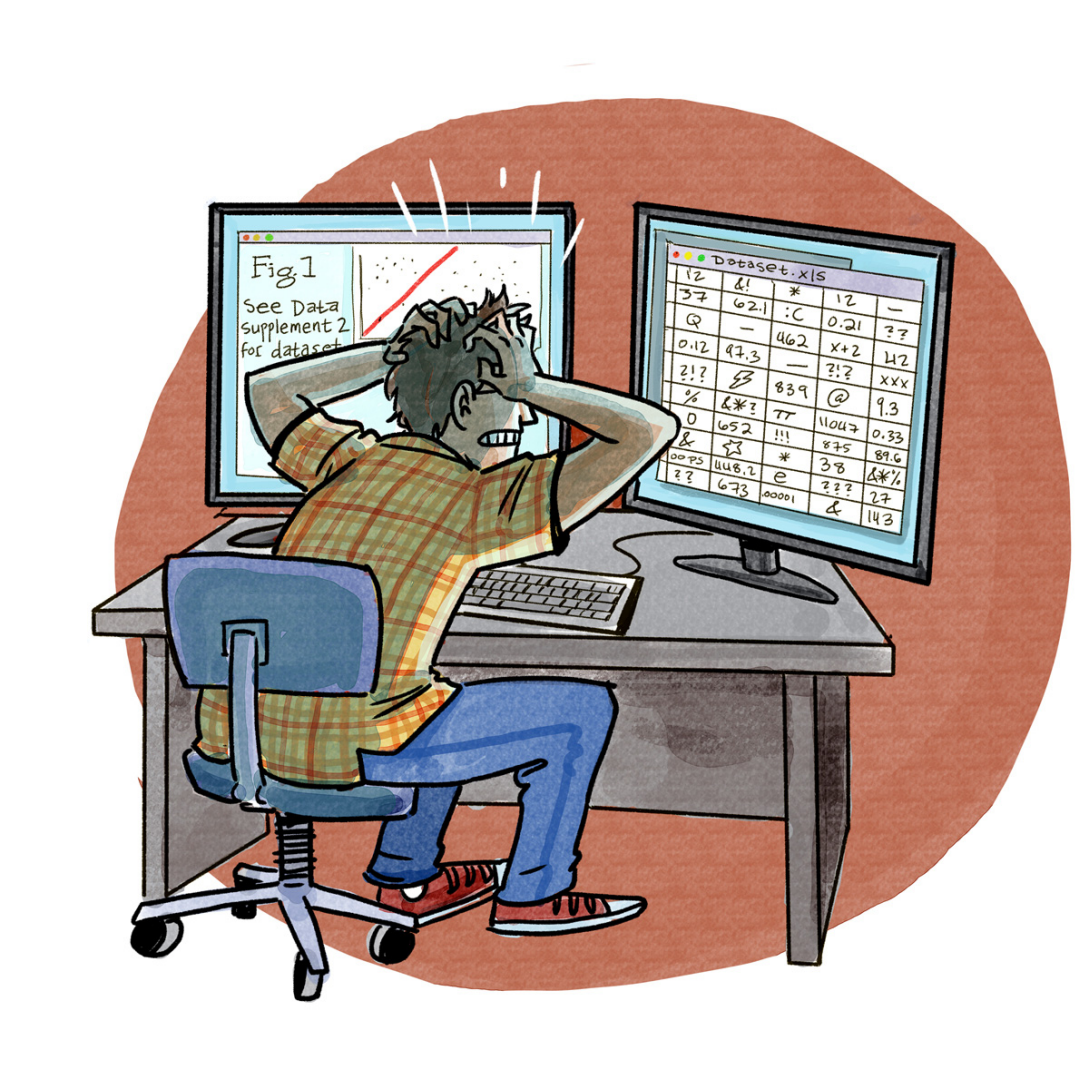
How complete and reusable are publicly archived data in ecology and evolution? The expectation of PDA that exists in genetics and molecular biology is rapidly permeating throughout ecology and evolution. With the advent of data archiving policies and integrated data repositories, journals and funders now have effective means of mandating PDA. However, the quality of publicly archived data associated with experimental and observational (nonmolecular) studies in ecology and evolution is highly variable. Illustration by Ainsley Seago.

**Table 1:** Journal and publication year of 100 reviewed studies with associated data publicly archived in the digital repository Dryad (http://datadryad.org/). At the time of data deposition in the repository, journals had either a “strong” PDA policy or adhered to the Joint Data Archiving Policy (JDAP), both of which require that data necessary to replicate a study’s results be archived in a public repository. Datasets were examined to assess completeness and reusability.

Journal	Policy	Number of Studies	
		2012	2013
*Biology Letters*	strong	2	10
*Evolution*	JDAP	16	13
*Evolutionary Applications*	JDAP	3	2
*Journal of Evolutionary Biology*	JDAP	17	10
*Nature*	strong	1	0
*Science*	strong	2	3
*The American Naturalist*	JDAP	9	12

**Table 2:** Data completeness and reusability assessment. Scoring system and criteria used to assess data completeness and reusability of 100 studies with data archived in the public repository Dryad.

Data Completeness		
Score	Description	Criteria
5	Exemplary	All the data necessary to reproduce the analyses and results (in practice) are archived. There is informative metadata with a legend detailing column headers, abbreviations, and units.
4	Good	All the data necessary to reproduce the analyses and results (in practice) are archived. The metadata are limited or absent, but column headings, abbreviations, and units can be understood from reading the paper.
3	Small omission	Most of the data necessary to repeat the analyses are archived except for a small amount (e.g., for a supporting or exploratory analysis). The metadata are informative OR the archived data can be interpreted from reading the paper.
2	Large omission	The main analyses in the paper cannot be redone because essential data are missing AND/OR insufficient metadata or information in the paper precludes interpreting the data AND/OR the authors archived summary statistics (e.g., means), but not the raw data used in the analyses.
1	Poor	The data are not archived OR the wrong data are archived OR insufficient information is provided in the metadata or paper for the data to be intelligible.
**Data Reusability**		
**Score**	**Description**	**Criteria**
5	Exemplary	The data are archived in a nonproprietary, human- and machine-readable file format that facilitates data aggregation and can be processed with both free and proprietary software (e.g., csv, text; see Table 3). The metadata are highly informative (such that column headings, abbreviations, and units can be understood in isolation from the original paper). Raw data are presented (perhaps in combination with processed data such as means).^a^
4	Good	The data are archived in a format that is designed to be machine readable with proprietary software (e.g., Excel), and the metadata are highly informative (such that column headings, abbreviations, and units can be understood in isolation from the original paper). [OR] The data are archived in a nonproprietary, human- and machine-readable file format, and the metadata are sufficiently informative to be understood when combined with information from the associated paper. Raw data are presented (perhaps in combination with processed data such as means).^a^
3	Average	The data are archived in a format that is designed to be machine readable with proprietary software (e.g., Excel). The metadata are sufficiently informative to be understood when combined with information from the associated paper. Raw data are presented (perhaps in combination with processed data such as means).^a^
2	Poor	The data are archived in a human- but not machine-readable format. The metadata are highly informative OR sufficiently informative to be understood with information from the associated paper. Raw data are presented (perhaps in combination with processed data such as means).^a^
1	Very poor	The metadata are insufficient for the data to be intelligible even when combined with information from the associated paper AND/OR processed but not raw data are presented.^a^

N.B. Reusability was assessed for archived data independently of completeness**.** One point was subtracted when data were included as supplementary material on the journal website, except when the reusability score was 1 to avoid zero values (see S1 Text).

^a^ Raw data were considered unprocessed data (e.g., trait values used in a principal component analysis rather than principle component scores, values underlying means presented in figures). Studies that did not archive duplicate or triplicate measurements to account for measurement error were not considered as missing raw data.

## How Well Are We Doing?

**Fig 2:**
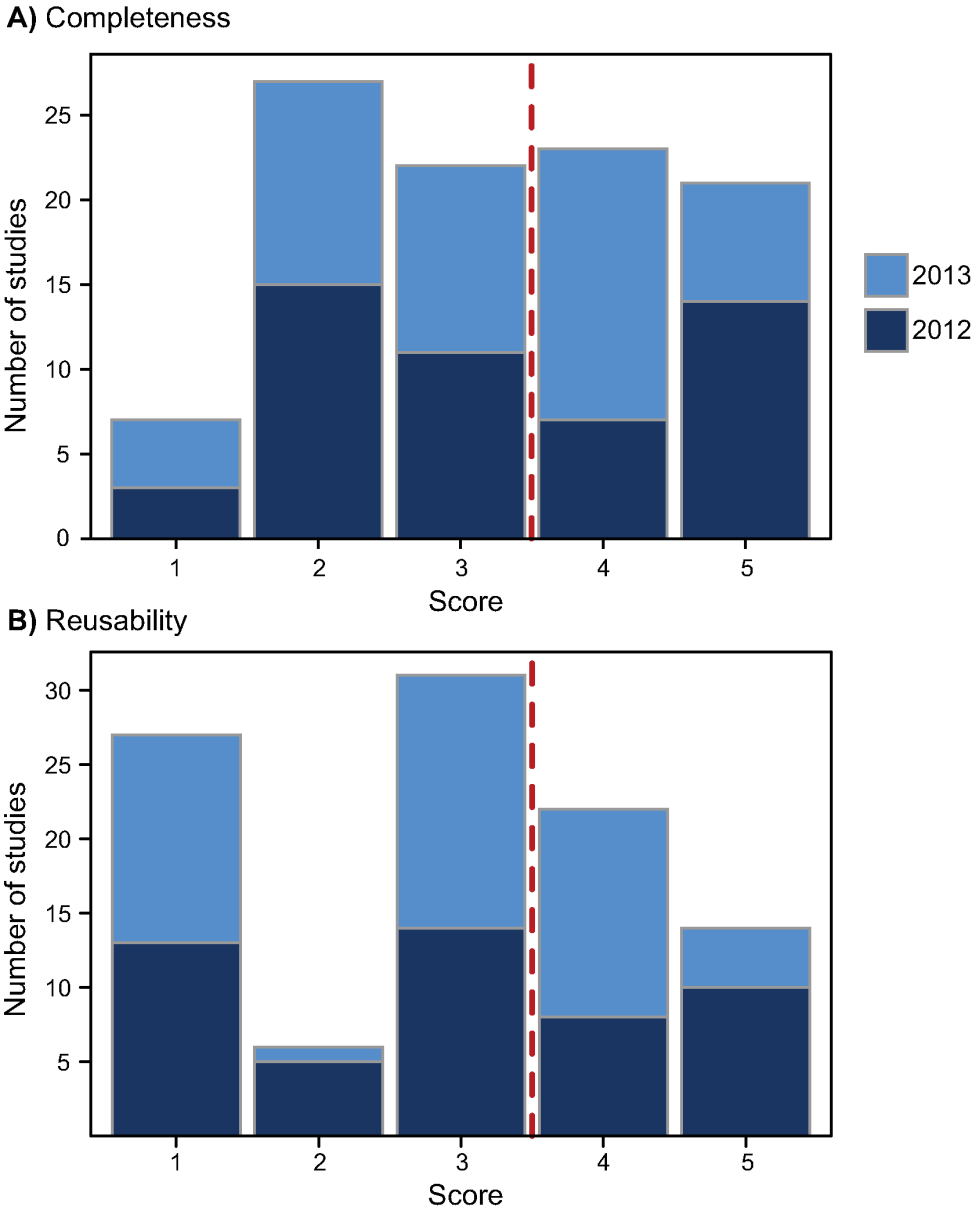
Completeness and reusability scores. Frequency distribution of public data archiving (PDA) scores for (A) completeness and (B) reusability across 100 studies in 2012 (light blue bars) and 2013 (dark blue bars). A score of 5 indicates exemplary archiving, and a score of 1 indicates poor archiving (see Table 2). Studies with completeness scores of 3 or lower (left of the red dashed line in panel A) do not comply with their journal's PDA policy. Studies to the left of the red dashed line in panel B have a reusability score between “average” (score of 3) and very poor (score of 1).

**Fig 3:**
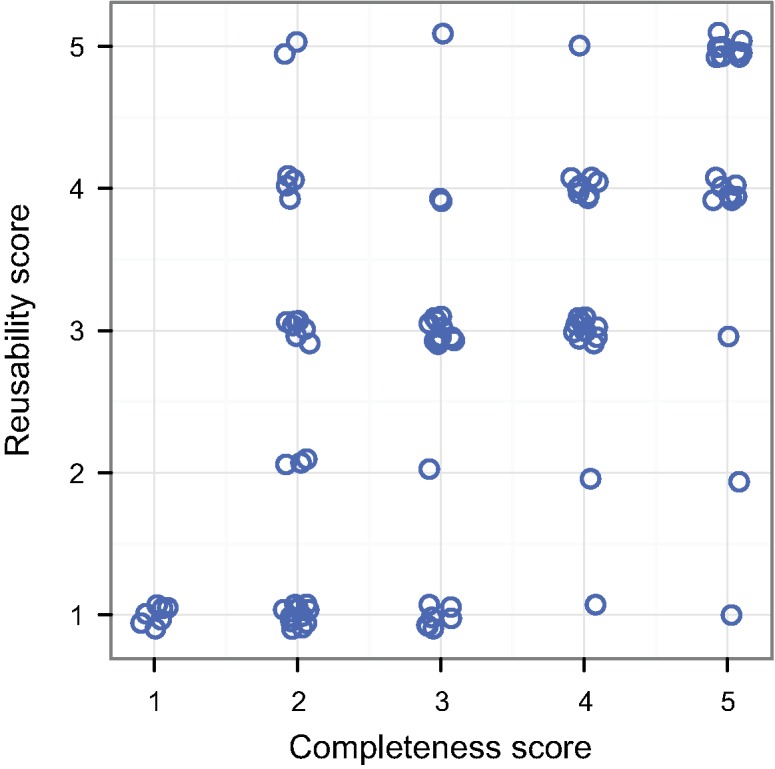
The relationship between the reusability and completeness of archived datasets (R = 0.59, *p* < 0.001). Empty circles are individual data points (offset to avoid overlap).

We found considerable variation in the quality of publicly archived data from the 100 studies surveyed, even though all were published either in JDAP journals or journals with a strong PDA policy. In most studies (56%), the archived datasets were incomplete, either because of missing data or insufficient metadata, resulting in a completeness score of 3 or less (Figs 1 and 2A). Therefore, these studies do not comply with the PDA policy of the journal in which they were published (Fig 2A), as strong policies (JDAP or other) require all the data supporting a paper’s results to be available in a public repository. Secondly, datasets for 64% of studies were archived in a way that either partially or fully prevented reuse (Fig 2B), either because they lacked essential metadata, because the data were presented in processed rather than raw form, or because inadequate file formats were used (e.g., non-machine-readable file formats, such as pdf, that require specialized software to read) (Fig 2B). Thus, even if these datasets could in theory be used to reproduce a study’s results, their value is questionable. Finally, there was a strong correlation between the completeness and reusability scores (Fig 3; R = 0.59 ± 0.07 SE, *p* < 0.001; see S3 Text for further details). In 22% of studies, some or all of the archived data were presented as electronic supplementary material. This is not ideal since, unlike files archived on Dryad, there are no standards for organizing supplementary data both within and across journals [37], and such data are often not readily discoverable or openly accessible (to those without a relevant journal subscription, for example) [33].

These findings are concerning given that (1) the studies were published in journals that enforce PDA, (2) our completeness score likely underestimates the number of irreproducible results since we did not attempt to replicate each study’s statistical analyses (see [9]), and (3) one key objective of PDA beyond increasing reproducibility is to accelerate scientific progress by facilitating data reuse [2,5,7]. Recent enforcement of PDA policies has had a positive effect on data deposition rates [22,23]. However, most journals do not verify the quality of archived data beyond basic checks such as ensuring that a data availability statement and a valid DOI are provided in the manuscript [38–40]. Therefore, datasets can contain involuntary errors and omissions [38]; we ourselves acknowledge errors made and possible improvements to past archived datasets.

Almost 40% of the 56 non-JDAP or non-journal policy compliant studies lacked only small amounts of data (completeness score of 3; Fig 2A). This suggests that many of these omissions are unintended and can be avoided with some slight improvements to data archiving practices. It is important to note, however, that authors concerned about potential individual costs of PDA (see [1,41–44]) can deliberately archive data to make them difficult or impossible for a third party to reuse (e.g., by archiving incomplete data or data in unusable formats) [12,17,41,45–47]. Notable examples have recently been pointed out on Twitter and other social media [41,48–50].

Many authors willingly participate in PDA because they believe in sharing data from publicly funded research, they wish to contribute to science beyond their own publications, and/or because they see individual benefits in doing so (e.g., increased citation rate [51], opportunities for coauthorship and new collaborations [1,2,7,20]). Despite these motivations, we uncovered a suite of problems that made understanding and assessing data difficult: omission of data necessary to reproduce results, nonexistent or insufficient data descriptors (e.g., no unit specifications or explanations of abbreviations and column headings in tables), inflexible file formats (e.g., “.sav” files that required the proprietary software SPSS Statistics to open), nonstandard data formats (e.g., colour coding of cells in tables, unspecified column headings), poor data organization (e.g., unclear tab labels for Excel documents with multiple spreadsheets, mismatches between column headings and variable labels in the associated paper, variable labels in a language other than English), and inclusion of poorly identified data unrelated to the paper (e.g., unspecified subsets of the data used for the analyses). The most common pitfalls that affected data reusability were inadequate metadata, the use of proprietary and non-machine-readable file formats (e.g., data tables archived as PDF and word documents; S1 Table, S2 Table), and failure to archive raw data (S3 Table).

Ecologists and evolutionary biologists receive little or no training in data management and may be unfamiliar with the best practices for proper data archiving (Table 3) [12,30,52]. The fact that a dataset’s completeness score was generally higher than its reusability score suggests that authors understand their obligation to share data but struggle to do this effectively (Fig 3, S3 Text). Small, simple improvements can dramatically increase the reusability of archived data with minimal time or monetary investments (e.g., [53,54]). We summarise key recommendations in Table 3. Based on our assessment of articles, we found that the datasets that had the highest completeness and reusability scores were often those in which the authors explicitly linked the archived data to figures and analyses in the paper. This simple practice greatly enhances the organization and interpretability of the data, enabling both authors and third parties to verify that all data points are present.

**Table 3:** Key recommendations to improve PDA practices. References listed provide specific details and more extensive discussion on these topics.

Recommendation	Description	Ref.
1. Be mindful of PDA	Plan for PDA before data collection so that data are well managed and prepared for deposition when a manuscript is submitted or published.	[2,18,20,55,56]
2. Make your data discoverable	Avoid archiving data as supplementary material. Use an established repository (e.g., figshare, Dryad, Knowledge Network for Biocomplexity (KNB), Zenodo)^a^.	[2,20,33,36,37,53,56]
3. Provide detailed metadata	Provide information about the data, including a description of column headings, abbreviations, units of measurement, and what figures and/or analyses the data correspond to. Other metadata can include how the data were collected and suggestions for how to best reuse them.	[2,12,18,20,33,40,47,53,56–58]
4. Use descriptive file names	Give data files names that are concise but indicative of their content. Avoid blank spaces.	[56,58]
5. Archive unprocessed data	As much as possible, share the data in their raw form. Provide both the raw and processed data used in the analyses.	[47,53,56,58]
6. Use standard file formats	Use file formats that are compatible with many different types of software (e.g., csv rather than excel files).	[18,20,33,37,47,53,56,58]
7. Facilitate data aggregation	Use existing standards whenever possible and deposit data in appropriate public databases (e.g., occurrence data in the Global Biodiversity Information Facility (GBIF), sequences in GenBank). Archive different types of data as distinct documents (not as multiple sheets in one document). Use standard table formats (columns for a variable type and rows for single observations), short variable names without spaces, and meaningful values for missing data (e.g., the abbreviation NA for “not applicable”). Avoid nested headers, merged cells, colour coding, footnotes, etc.	[12,18,20,28,47,53,56,59]
8. Perform quality control	Check the format (e.g., numeric versus string) and units of values in a table. Ask a colleague to review the data and metadata for completeness and clarity.	[2,18,53,56]
9. Chose a publishing license	Use well-established licences (e.g., Creative Commons licenses^b^) to determine the responsibilities of reusers. The Creative Commons Zero licence (CC0) places no restrictions on data reuse and is preferred by many repositories.	[7,21,33,53,56]
10. Decide on an embargo	By default, data repositories release archived datasets immediately or upon publication of the associated paper. Some journals and repositories allow a one-year no-questions-asked embargo^c^. Longer embargos can be granted but require a special agreement with editors.	[1,2,21,33,36,55]

^a^ See Table 1 in [32,33] for further details and examples of recognized data repositories. Some repositories are free (e.g., figshare), and others have a data publishing charge [60]. Depending on the publishing journal, charges may be covered (http://datadryad.org/pages/integratedJournals).

^b^ http://creativecommons.org/

^c^ E.g., Dryad allows a one-year no-questions-asked embargo, but figshare offers no embargo option.

## Which Way Forward?

Participation in PDA is on the rise, but its benefits require that authors archive complete and reusable datasets. Suggestions to improve acceptance of PDA policies are diverse and include treating data associated with journal articles as formal publications (i.e., publish data papers) [6,20,40,61,62], providing incentives for best practices so that authors voluntarily archive high-quality, reusable data [2,7,28,53], and allowing reasonable embargoes for researchers who have planned further uses for their data [1,19,21,36]. Obviously, increased policing of publicly archived datasets by journals and/or archive curators (i.e., reviewing archived data) should also increase the quality of archived data [22,24,38,45,63]. All of these recommendations have merit, but it is unlikely that there is one ideal solution.

From a practical point of view, enforcing PDA on unwilling authors is largely ineffective because cheating is easy—trying to reproduce the results of every submitted manuscript is virtually impossible. Publishing data papers is a valid solution for large, important datasets with a high reuse potential [40,64], but there are good reasons to think that this model is both impractical and unlikely to succeed for data that underlie most publications [62], namely because many datasets are limited in their size, scope, and/or novelty, which might not warrant publication in a data journal [40,61]. Reviewers and editors are also already overloaded with article peer reviews, almost always without compensation from publishers. Therefore, additional requests to police data associated with traditional papers could be perceived as unreasonable [6]. Finally, data repositories currently lack the funding to perform thorough technical reviews to verify that datasets and metadata are complete and concordant with the information in a paper [6,36]. For example, Dryad is currently forced to charge archiving fees to operate [60] but only has enough curators to perform basic checks on data submissions such as verifying that files can be opened and are free of viruses [65].

Rather than punishing researchers who do not share their data, there are strong arguments for rewarding those who do [1,66,67]. This idea is in line with recent calls for a culture shift towards more collaboration in science [68,69], in which the value and importance of PDA is emphasized and greater benefits given to active participants [1,12,31,33,63]. These benefits can take many forms, including credit from hiring or promotion committees and funding agencies [12], as well as prizes from departments, societies, and publishers for most reusable or reused dataset, best data paper, or most reproducible results [63]. An important move in this direction was the 2013 San Francisco Declaration on Research Assessment (DORA), which recommends considering datasets and other types of scientific contributions (e.g., software, training) when scientists’ research outputs are evaluated [70].

Importantly, sociological studies (both experimental and theoretical) point to the fact that both “sticks” and “carrots” are necessary to improve cooperation [71,72]. A recent theoretical study of a public good game, a standard framework for cooperation in groups, showed that the policy “first carrot, then stick” is highly successful at promoting cooperation because it combines the effectiveness of rewarding to establish cooperation with the effectiveness of punishing to maintain it [72]. Those who comply must first be rewarded, and, once compliance has become the norm, it can become mandatory and enforced by a penalty for noncompliance [72]. This strategy has major advantages for PDA in that offering “carrots” can shift the culture to the point at which authors publicly archive their data even when they are not required to do so [12].

## Conclusion

Our results suggest that at least some parts of public data archives are being used to maintain datasets in E&E that are of little use for reproducing existing studies or carrying out new ones. These findings, combined with those of the few other studies that have also explored this issue [9,27], suggest that the problem is ubiquitous, touching both molecular and nonmolecular fields of biology. Clearly, improvements to current PDA practices are necessary. Solutions might not be straightforward, but they may have to include strategies combining enforcement, reward, and flexibility [1]. Importantly, PDA is quite new for ecologists and evolutionary biologists, and our results indicate that substantial improvements to its value can be made with relatively little effort.

### Data Availability

The data and code for this study are available on the repository figshare: http://dx.doi.org/10.6084/m9.figshare.1393269.

### Data Reuse

The list of publications with associated data archived in Dryad from inception to 20 Sep 2013 was kindly compiled and publicly archived by Vision et al. [73].

## Supporting Information

S1 Table Terminology used to describe data file formats. (DOCX)

S2 Table Characteristics (nonproprietary, human readable, machine readable) of archived file formats encountered in this study. 0 = no, 1 = yes. A greater row total indicates a higher reuse potential (NA was treated as a 1).
(DOCX)

S3 Table Summary of information contained in the public dataset associated with this study. Number of datasets (out of 100) that (1) have a useful readme file, (2) are archived in nonproprietary machine- and human-readable file formats, (3) were analysed with a statistical program that allows scripting/coding, (4) have associated analysis code publicly archived, (5) were analysed with an statistical program that is not specified in the publication. Mean completeness and reusability scores across the 100 datasets were examined.
(DOCX)

S1 Text Materials and methods. (DOCX)

S2 Text Interrater agreement analysis. (DOCX)

S3 Text Results: Relationship between reusability and completeness. (DOCX)

## Abbreviations

CC0: Creative Commons Zero licence
DORA: Declaration on Research Assessment
E&E: ecology and evolution
GBIF: Global Biodiversity Information Facility
JDAP: Joint Data Archiving Policy
KNB: Knowledge Network for Biocomplexity
PDA: public data archiving
