# Supplementary material for: Public Data Archiving in Ecology and Evolution: How Well Are We Doing?
Source: PLoS Biol. 2015 Nov 10;13(11):e1002295. doi: 10.1371/journal.pbio.1002295 (PMC4640582; doi:10.1371/journal.pbio.1002295)
Supplement: S1 Table — (DOCX) [file pbio.1002295.s001.docx]

**S1 Table**

| Proprietary | Format designed for use with commercial software. |
| --- | --- |
| Non-proprietary | Format designed for use with a variety of different freely-available software. |
| Machine-readable | Format that allows data to be read by a computer (e.g. csv but not PDF files). |
| Human-readable | Format that allows data to be read by humans (e.g. csv and PDF files). |
