# Supplementary material for: Public Data Archiving in Ecology and Evolution: How Well Are We Doing?
Source: PLoS Biol. 2015 Nov 10;13(11):e1002295. doi: 10.1371/journal.pbio.1002295 (PMC4640582; doi:10.1371/journal.pbio.1002295)
Supplement: S2 Table — 0 = no, 1 = yes. A greater row total indicates a higher reuse potential (NA was treated as a 1). (DOCX) [file pbio.1002295.s002.docx]

**S2 Table.**

| **File extension** | **Non-proprietary** | **Human-readable** | **Machine-readable** | **Total** |
| --- | --- | --- | --- | --- |
| .avi | 0 | 1 | NA | 2 |
| .csv | 1 | 1 | 1 | 3 |
| .doc | 0 | 1 | 0 | 1 |
| .docx | 0 | 1 | 0 | 1 |
| .mp4 | 0 | 1 | NA | 2 |
| .nex | 1 | 1 | 1 | 3 |
| .pdf | 0 | 1 | 0 | 1 |
| .rtf | 0 | 1 | 0 | 1 |
| .sas | 0 | 1 | 1 | 2 |
| .sav | 0 | 1 | 1 | 2 |
| .txt | 1 | 1 | 1 | 3 |
| .wav | 0 | 1 | 0 | 1 |
| .xls | 0 | 1 | 1 | 2 |
| .xlsx | 0 | 1 | 1 | 2 |
| .xml | 1 | 1 | 1 | 3 |
