# Supplementary material for: Public Data Archiving in Ecology and Evolution: How Well Are We Doing?
Source: PLoS Biol. 2015 Nov 10;13(11):e1002295. doi: 10.1371/journal.pbio.1002295 (PMC4640582; doi:10.1371/journal.pbio.1002295)
Supplement: S3 Table — Number of datasets (out of 100) that (1) have a useful readme file, (2) are archived in nonproprietary machine- and human-readable file formats, (3) were analysed with a statistical program that allows scripting/coding, (4) have associated analysis code publicly archived, (5) were analysed with an statistical program that is not specified in the publication. Mean completeness and reusability scores across the 100 datasets were examined. (DOCX) [file pbio.1002295.s003.docx]

**S3 Table**

|  | No. datasets |
| --- | --- |
| Complete readme file ^a^ | 40 |
| Non-proprietary, machine- and human-readable file format | 38 |
| Statistical analysis program allows scripting/coding | 75 |
| Statistical analysis code publicly archived | 4 |
| Statistical analysis program not specified | 18 |
| Data not archived but included as electronic supplementary material ^b^ | 22 |
|  | Mean |
| Completeness score | 3.24 |
| Reusability score | 2.90 |

^a^ Information in the readme file or Dryad data description box is sufficient to make sense of the archived data (i.e. understand the column headings, abbreviations and units) without having to refer to the paper.

^b^ Some data are included as electronic supplementary material but not archived on Dryad.
