# Supplementary material for: Public Data Archiving in Ecology and Evolution: How Well Are We Doing?
Source: PLoS Biol. 2015 Nov 10;13(11):e1002295. doi: 10.1371/journal.pbio.1002295 (PMC4640582; doi:10.1371/journal.pbio.1002295)
Supplement: S1 Text — (DOCX) [file pbio.1002295.s004.docx]

**S1 Text**

We randomly selected 100 studies from a recently compiled list of published papers that have their associated data archived in the public repository Dryad [1]. Dryad is a general, online repository for evolutionary and ecological data established concurrently with, and recommended by, JDAP [2]. In total, data from seven journals were assessed (Table 1). We excluded studies published in non-JDAP journals and in journals without a strong PDA policy. We also excluded meta-analyses, studies which had a strong theoretical or modelling component, or which primarily used molecular or genetic techniques to answer their research questions. We evaluated 50 studies published in 2012 and 50 published in 2013. We carefully read and assessed each paper, the archived data files and any associated electronic supplementary material, noting details of all the variables used in the figures, tables and analyses.

***Data completeness***

We evaluated data *completeness* based on two criteria: 1) whether sufficient descriptors were provided to make sense of the archived data and 2) whether all the necessary data were available to reproduce the analyses, tables, and figures in the published manuscript. We did not attempt to contact authors to obtain missing data or data descriptors because our aim was to evaluate the quality of the archived data. The objective of JDAP is to ensure long-term data preservation by avoiding e-mail and hyperlink rot (e.g. web pages becoming permanently unavailable) [3,4] and allow data reuse without having to obtain consent from the original authors. We did not penalize studies for not archiving raw data as long as the analyses and the figures presented in the article could be reproduced, in theory, with the data made available. For example, for the completeness score, principal component values were deemed sufficient if these values rather than the raw data were used in the analyses and/or presented in the figures. Due to the large diversity in the types of data, analyses and statistical programs used, we did not attempt to explicitly reproduce each study’s statistical results from the original data [see 5,6]. As such, our findings likely overestimate the extent to which published results can be reproduced [see 5].

We assigned each study a completeness score between 1 and 5; the criteria associated with each mark are outlined in Table 2. Studies scoring a 5 represented exemplary data archiving, meaning that the data were complete and archived with informative metadata. Studies with scores of 3 or less were considered ‘non-compliant’ with the reproducibility requirement of JDAP and other strong PDA policies.

***Data reusability***

We assessed data *reusability* independently of completeness (i.e. only for archived data). In addition to information previously collected, we recorded the format of the archived files (e.g. text, Excel, pdf) and the type of values or measurements they contained (i.e. raw vs. processed). We used three main criteria to assign studies a reusability score between 1 and 5: 1) whether sufficient descriptors were provided to make sense of the archived data (same as for data completeness); 2) whether the data were archived in a non-proprietary, human- and machine-readable file format that facilitates data aggregation and could be processed with both free and non-proprietary software (e.g. csv, text; Tables S1, S2); and 3) whether raw rather than processed data were archived (e.g. trait values used in a principal component analysis rather than PC scores, which have a much lower reuse potential, even if they scored highly for completeness above).

Studies with informative metadata, modifiable file formats compatible with many different software types, and with unprocessed data were considered highly reusable (score of 5). Studies scored lower if they lacked essential metadata, archived data in a format designed to be machine-readable with proprietary software (e.g. Excel), non-machine-readable (e.g. pdf), or if the archived data were processed. A detailed description of each score is provided in Table 2. One point was subtracted if some data were only included as electronic supplementary material on the journal website (rather than on Dryad) because this reduces data discoverability and, in many cases, prevents access without a journal subscription [2,6]. We reiterate that sufficient reusability is not an explicit requirement of journal PDA policies – therefore, a low reusability score does not necessarily constitute lack of compliance with a journal’s PDA policy.

***Analysis***

We assessed interrater agreement using Krippendorff’s alpha (α) [7,8], which ranges from 1 indicating perfect agreement to 0 indicating the absence of agreement. Ten papers were randomly selected and independently examined by four people (S2 Text). Interrater agreement was high for both data completeness (α = 0.79) and reusability (α = 0.86) (S2 Text). These values should be interpreted with caution, however, because these 40 assessments (4 raters x 10 papers each) represent 10% of the total datasets examined in our study. Additionally, all four raters are familiar with the data structures and types of data that ecologists and evolutionary biologists typically collect. Hence, it is possible that raters with different areas of expertise would have a different understanding of the same data, resulting in lower interrater agreement scores.

To test whether completeness and reusability scores differed between years or journals and to simultaneously estimate the correlation between the two scores, we ran a bivariate model using the R package ASReml-R [9]. We tested for differences in completeness and reusability with a Wilcoxon signed-rank test. Analyses were done in R.3.0.2 [10].

The data and code for this study are publicly available on the repository figshare (http://dx.doi.org/10.6084/m9.figshare.1393269); authors’ names and DOI links are not given in the table, but researchers can request a key to verify our results. As stated by others [11], the issues presented in this paper are ubiquitous and should not be used to question the integrity of individual researchers, especially as we aim to evaluate issues that may not have occurred to authors when depositing their data.

**References**

1. Vision TJ, Scherle R, Mannheimer S. Data for: Embargo selections of Dryad data authors. figshare. 2013. Available from: http://dx.doi.org/10.6084/m9.figshare.805946.

2. Vision TJ. Open data and the social contract of scientific publishing. BioScience. 2010, 60: 330-331. doi: 10.1525/bio.2010.60.5.2.

3. Teunis T, Nota SP, Schwab JH. Do corresponding authors take responsibility for their work? A covert survey. Clin Orthop Relat Res. 2015, 473: 729–735. doi: 10.1007/s11999-014-3868-3.

4. Costello MJ, Michener WK, Gahegan M, Zhang Z-Q, Bourne PE. Biodiversity data should be published, cited, and peer reviewed. Trends Ecol Evol. 2013, 28: 454-461.

5. Gilbert KJ, Andrew RL, Bock DG, Franklin MT, Kane NC, Moore JS, et al. Recommendations for utilizing and reporting population genetic analyses: the reproducibility of genetic clustering using the program structure. Mol Ecol. 2012, 21: 4925-4930.

6. Caetano DS, Aisenberg A. Forgotten treasures: the fate of data in animal behaviour studies. Anim Behav. 2014, 98: 1-5. doi: http://dx.doi.org/10.1016/j.anbehav.2014.09.025.

7. Hayes AF, Krippendorff K. Answering the call for a standard reliability measure for coding data. Commun Methods Meas. 2007, 1: 77-89.

8. Krippendorff K. Estimating the reliability, systematic error and random error of interval data. Educ Psychol Meas. 1970, 30: 61-70.

9. Butler DG, Cullis BR, Gilmour AR, Gogel BJ. ASReml-R reference manual 2009. Available from: http://www.vsni.co.uk/downloads/asreml/release3/asreml-R.pdf.

10. R Development Core Team. R: A language and environment for statistical computing. R Foundation for Statistical Computing, Vienna, Austria, ISBN 3-900051-07-0. 2014.

11. Magee AF, May MR, Moore BR. The dawn of open access to phylogenetic data. PLoS One. 2014, 9: e110268. doi: 10.1371/journal.pone.0110268.
