# Supplementary material for: Public Data Archiving in Ecology and Evolution: How Well Are We Doing?
Source: PLoS Biol. 2015 Nov 10;13(11):e1002295. doi: 10.1371/journal.pbio.1002295 (PMC4640582; doi:10.1371/journal.pbio.1002295)
Supplement: S2 Text — (DOCX) [file pbio.1002295.s005.docx]

**S2 Text**

We estimated a measure of repeatability of our scoring system by examining interrater reliability/agreement on a subset of the 100 papers in our analysis. Ten papers were randomly selected and independently examined by four people, all with evolutionary or ecological interests. The four raters included the two authors who collected the data for the study, DGR and SAB (raters A and B), and two independent researchers who are not authors on the paper and were hired for this purpose (raters C and D). Raters C and D were given a document with instructions detailing how to access the studies and associated datasets and then assess the completeness and reusability of archived data following the Methods as described in S1 Text and Table 2; see section “Protocol for assessing archived data completeness and reusability” below for the instructions provided.

We assessed interrater agreement using Krippendorff’s alpha coefficient (α), which measures score agreement between different raters [1,2]. Krippendorff’s alpha ranges from 1 indicating perfect agreement to 0 indicating the absence of agreement (i.e. scores are statistically unrelated to the units they describe). Unlike other measures of interrater agreement (e.g. Cohen’s kappa, Fleiss’s Kappa), this coefficient can accommodate data that are ordinal *and* collected by more than two observers [1]. We assessed interrater agreement between the two primary data collectors (raters A and B; hereafter denoted as α_2_) and among all four raters (raters A, B, C, D; hereafter denoted as α_4_) on an ordinal (score from 1 to 5) and nominal (pass = score > 3, fail = score <3) scale. The code and data for these analyses are archived on figshare (http://dx.doi.org/10.6084/m9.figshare.1393269).

**Results**

Interrater agreement was high for both data completeness (α_2_ = 0.91, α_4_ = 0.79) and reusability (α_2_ = 0.95, α_4_ = 0.86) scores assigned by either two or four raters (i.e. on an ordinal scale). Discrepancies in scores assigned by the different raters resulted in few disagreements over whether a study passed or failed these two criteria. There was a single case of disagreement between the two primary raters over whether the study passed the reuse criterion, and no disagreement between these raters over the completeness criterion. There were only two instances for completeness and three for reuse where one of the four raters’ scores placed a study in a different category (pass/fail) than the other three. Therefore, interrater agreement was also high for data completeness (α_2_ = 1.00, α_4_ = 0.78) and reusability (α_2_ = 0.81, α_4_ = 0.70) on a nominal scale.

**Protocol for assessing archived data completeness and reusability**

1. The 10 studies/datasets to assess are listed in the file “X_Repeatability_analysis.xlsx”, where X is you rater number.

2. For each study, download: 1) the pdf and associated supplementary material from www.doi.org, and 2) the archived data files from <http://datadryad.org/>. Enter the paper’s doi rather than the Dryad doi in Dryad to access all the files associated with the paper. Contact me (dominique.roche@mail.mcgill.ca) if you do not have access to the journal. Save the files in a specific folder for each study. Readme files are usually in a data description box on Dryad or as a separate, archived text file.

3. Follow the methods described in the manuscript. Use the criteria in Table 2 of the manuscript to assign each study a data completeness and reusability score, noting justifications in the ‘notes’ column of the “X_Repeatability_analysis.xlsx” file. Fill in the different columns in the “X_Repeatability_analysis.xlsx” file, referring to the metadata tab for a description of each column’s content.

4. Five examples are listed under the third tab of the “X_Repeatability_analysis.xlsx” file.

**References**

1. Hayes AF, Krippendorff K. Answering the call for a standard reliability measure for coding data. Commun Methods Meas. 2007, 1: 77-89.

2. Krippendorff K. Estimating the reliability, systematic error and random error of interval data. Educ Psychol Meas. 1970, 30: 61-70.
