# Supplementary material for: Public Data Archiving in Ecology and Evolution: How Well Are We Doing?
Source: PLoS Biol. 2015 Nov 10;13(11):e1002295. doi: 10.1371/journal.pbio.1002295 (PMC4640582; doi:10.1371/journal.pbio.1002295)
Supplement: S3 Text — (DOCX) [file pbio.1002295.s006.docx]

**S3 Text**

There was a strong correlation between the completeness and reusability scores (R = 0.593 ± 0.068 SE, *P* < 0.001) but no indication of differences between years (completeness: Wald statistic = 0.10, d.f. = 1, *P* = 0.754; reusability: Wald statistic = 0.02, d.f. = 1, *P* = 0.902) or journals (completeness: Wald statistic = 3.90, d.f. = 6, *P* = 0.691; reusability: Wald statistic = 3.35, d.f. = 6, *P* = 0.764). On average, the completeness of datasets was higher than their reusability (completeness: 3.24 ± 1.26; reusability: 2.90 ± 1.39; mean ± SD; V = 1311.5, *P* = 0.0061), and there was considerable variation in reusability even for datasets with high completeness scores.
